# A Bi-Functional Targeted P28-NRC Chimeric Protein with Enhanced Cytotoxic Effects on Breast Cancer Cell Lines

**DOI:** 10.22037/ijpr.2019.2392

**Published:** 2019

**Authors:** Meysam Soleimani, Hamid Mirmohammmad Sadeghi, Ali Jahanian-Najafabadi

**Affiliations:** a *Department of Pharmacognosy and Pharmaceutical Biotechnology, School of Pharmacy, Hamadan University of Medical Sciences, Hamadan, Iran.*; b *Department of Pharmaceutical Biotechnology, School of Pharmacy and Pharmaceutical Sciences, Isfahan University of Medical Sciences, Isfahan, Iran.*; c *Isfahan Pharmaceutical Sciences Research Center, School of Pharmacy and Pharmaceutical Sciences, Isfahan University of Medical Sciences, Isfahan, Iran.*

**Keywords:** Breast Cancer, Chimeric proteins, Azurin-p28, Antimicrobial Cationic peptides

## Abstract

One of the emerging therapeutic strategies for targeted therapy of cancer is the use of chimeric proteins. The p28 peptide has the ability of selective entrance and activating apoptosis in breast cancer cells. The NRC antimicrobial peptide showed cytotoxic activity on various breast cancer cell lines including drug-resistant cells and also on normal cells. Here we designed a chimeric protein consisting of these peptides to determine their targeted effects and to enhance their cytotoxic effects on breast cancer cells. The chimeric protein was cytotoxic to MDA-MB-231 and MCF7 breast cancer cell lines in a dose-dependent manner after 48 h of treatment. In addition, the cytotoxic effects of the p28 alone were significantly lower than the chimeric protein indicating the additive or enhanced effects of the two peptides. Flow cytometry analysis showed that the induced cell death is mediated via apoptosis. The designed chimeric protein had enhanced effects on breast cancer cell lines and exerted its anticancer effects on MCF7 breast cancer cells through mitochondrial caspase dependent and -independent apoptotic pathways. Taken together, the results of this study suggested the chimeric protein to be a reasonable anti-cancer agent which must be further evaluated by subsequent *in-vitro* and *in-vivo* preclinical studies.

## Introduction

It has been shown that forty percent of breast cancer patients have occult metastases at the time of diagnosis (1). The most common method for treatment of metastatic cancer is chemotherapy (2). However, a series of limitations restrict the application of chemotherapeutics, including the destruction of healthy cells in addition to cancer cells, inefficiency on dormant and slow-growing cells, secondary malignancies, and development of chemoresistant cancer cells (3-5). Anticancer therapy aims to find an approach to selectively kill cancer cells and restrict side effects to the minimum, unaffected by common mechanisms of chemoresistance, and kill dormant and slow-growing cells (6). A growing number of studies have shown that peptides can be a good candidate for the production of new medications for cancer treatment. Peptides can be divided into several functional categories in order to provide a targeted anti-cancer agent: killing peptides, which kill cancer cells by different mechanisms; cell penetrating peptides, which mediate transportation through cell membrane; targeting peptides, which cause targeted transmission toward cancer cells; and peptides which intervene in protein-protein interactions involved in apoptosis (7, 8). The combination of a targeting peptide with a killer peptide makes a peptide fusion which can be considered as a targeted-killer chimeric protein. In fact, this chimeric protein has two functional domains: homing peptide which could selectively bind to cancer cells, and a killing peptide which leads to the destruction of cancer cells. 

Azurin, a protein secreted by the *Pseudomonas aeruginosa *is a copper-containing redox protein (cupredoxin) (9). Various studies reported that a fragment of azurin protein, called p28, has the ability of selective entrance and activation of apoptosis in breast cancer cells (10). Following internalization, this peptide is able to inhibit cancer cells proliferation by stabilizing p53. This results in an increase in intracellular levels of p53 and impedes cell cycle in G2-M. Furthermore, treatment of immunodeficient nude mice with this peptide showed a reduction in the incidence of measurable tumors when compared to the control groups (11). 

NRC-03, a pleurocidin family of cationic antimicrobial peptides (CAPs), is also cytotoxic to multiple breast cancer cell lines. Furthermore, intratumoral administrations of NRC-03 killed breast cancer cells in xenografts in severe combined immunodeficiency (SCID) mice. Mitochondrial membrane damage is a mechanism that has been identified for the destruction of cancer cells by this peptide (12). 

The p28 peptide has entered a clinical trial for the treatment of recurrent or progressive central nervous system tumors and advanced solid tumors (ClinicalTrials.gov). However, the high mutation rate in p53 of human tumors may interfere with the interaction between p28 and p53, and compromise its efficacy on cancer treatment. Heterogeneity of tumors is another issue that affects its potential for cancer therapy. On the other hand, NRC peptide kills human tumor cells in a p53-independent manner as a concentration-dependent (12). Therefore, in this study, we developed a chimeric protein containing p28 as a homing/killer peptide and NRC as a killer peptide through recombinant technologies to target and potentiate the cytotoxicity of both moieties. Then, following expression and purification of the chimeric protein, its potency against breast cancer cells was examined. 

## Experimental

*General*

All cell lines used in the experiments were obtained from National Cell Bank of Iran (Pasteur Institute, Iran). The chimeric protein coding sequence and the coding sequence for p28 peptide were de novo synthesized by Nedayefan Company (Tehran, IRAN) and supplied in pGE plasmid. Glutathione Resin was purchased from GenScript (MA, USA) and Mouse anti-GST antibody was purchased from Abcam (Abcam, USA). FastDigest ^TM^ *Bam*HI, *Sal*I, *Eco*RI, and *Xho*I restriction endonucleases and also T4 DNA ligase was purchased from Thermoscientific (USA). The cells were cultivated in RPMI and DMEM 1640 containing 10% FBS and supplemented with 100 U/mL penicillin and 100 mg/mL streptomycin (Sigma, Germany). Amicon filters were obtained from Merck (Merck Millipore, USA).

*Construction of recombinant plasmids, expression, and purification of the p28-NRC chimeric protein*

The coding sequence of the chimeric protein and the p28 peptide was sub-cloned in the pGEX-5X-1 expression plasmid. This plasmid adds GST tag to the N-terminus of the expressed protein resulting in higher solubility and also aiding in affinity purification of the recombinant protein by glutathione resin. The fidelity of the cloning was confirmed by restriction enzyme digestion and subsequent DNA sequencing. The expression was investigated at various concentrations of IPTG, temperatures, and induction time. Glutathione Resin was packed in an empty column and was used for purification of GST tagged-chimeric protein and the p28 peptide using fast protein liquid chromatography (FPLC). In order to purify the expressed protein, the IPTG-induced bacterial cells were collected by centrifugation and resuspended in phosphate-buffered saline (PBS). Then, the mixtures were sonicated on ice and subsequently centrifuged at 10,000 × g for 15 min. The collected supernatant was used for the purification of GST-tagged chimeric protein and the p28. Elution of GST-tagged chimeric protein and p28 from chromatography medium was performed under mild, non-denaturing conditions using reduced glutathione. Then, the fractions were collected and analyzed by SDS-PAGE and Western blotting. Fractions containing the chimeric protein or p28 were pooled and subjected to dialysis followed by enterokinase cleavage of the GST-tag. When the GST tags removed, protein samples were concentrated using Amicon™ filters. Finally, gel filtration chromatography by Superdex 75 (GE Healthcare, USA) was used to achieve highly pure recombinant protein or p28 peptide suitable for upcoming *in-vitro* and *in-vivo* studies.

*Cytotoxicity Assay*

Cytotoxicity of the chimeric protein was assessed by MTT assay. To measure cytotoxicity, the cells were cultivated in 96 well plates and treated with various concentrations of chimeric protein or p28 peptide for 48 h. Then, the medium was replaced with fresh medium containing 5 mg/mL MTT and further incubated for 4 h. Afterward, the supernatant was replaced with 150 µL Dimethyl sulfoxide (DMSO) to dissolve the formazan crystals and finally, absorbance was read at 450 nm with a microplate reader. Results were calculated as the percent of the absorbance of the treated cells over the absorbance of the untreated control cells. Based on these measurements, the IC_50_ values of chimeric protein and p28 peptide were calculated.

**Figure 1:**
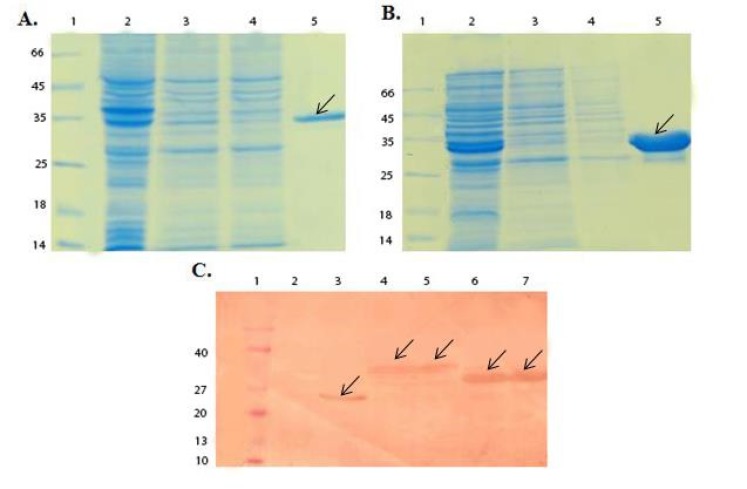
SDS-PAGE and Western blot analysis of GST-chimeric protein and GST-p28 purification. (A) SDS-PAGE analysis of GST- chimeric protein purification. Lane 1; Molecular weight protein marker (26610, Fermentase), Lane 2; Supernatant before applying to the column, Lane 3 and 4; Supernatant after applying to the column, Lane 5; Elution of GST- chimeric protein with 10 mM glutathione. The theoretical MW of the GST-p28 is about 36.5 kDa. Arrow indicated the purified GST-chimeric protein. (B) SDS-PAGE analysis of GST-p28 purification. Lane 1; Molecular weight protein marker26610) , Fermentase), Lane 2; Supernatant before applying to the column Lane 3; Supernatant after applying to the column Lane 4; GST-p28 after elution with the reduced glutathione. The theoretical MW of the GST-p28 is about 32.6 kDa. (C) Western blot analysis of GST, GST-chimeric protein, and GST-p28. Lane 1; Molecular weight protein marker (BM0066, FMC.Bioproducts), Lane 2; Uninduced pGEX, Lane 3; Induced pGEX, Lane 4 and 5; Induced GST- chimeric protein, Lane 6 and 7; Induced GST-p28

**Figure 2:**
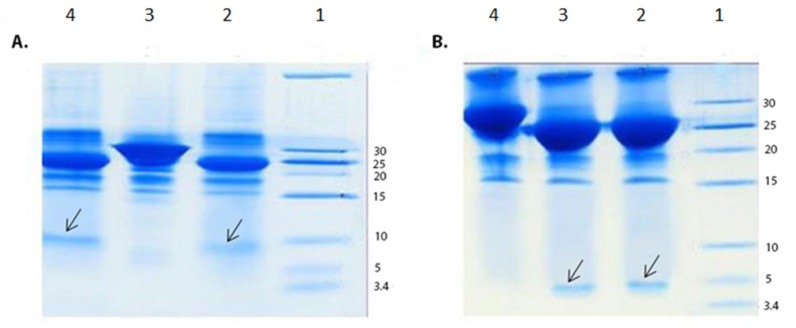
SDS-PAGE analysis of enterokinase digestion of the GST-chimeric protein and GST-p28 peptide. (A) SDS-PAGE analysis of enterokinase digestion of the GST-chimeric protein. Lane 1; Molecular weight marker Lane 2; Digested GST- chimeric protein using 1 unit of the enterokinase Lane 3; Undigested GST-chimeric protein Lane 4; Digested GST-chimeric protein using 2 units of the enterokinase. Arrow indicated the chimeric protein. The theoretical MW of the chimeric protein is about 7 kDa. (B) SDS-PAGE analysis of enterokinase digestion of the GST-p28 Lane 1; Molecular weight marker Lane 2; Digested GST-p28 with 1 unit of enterokinase Lane 3; Digested GST-p28 with 2 units of enterokinase Lane 4; Undigested GST-p28. Arrow indicated the p28 peptide. The theoretical MW of the p28 peptide is about 3 kDa

**Figure 3:**
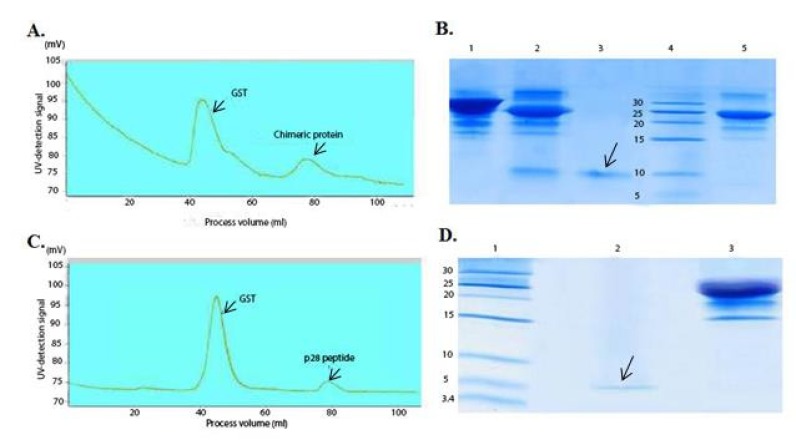
Chromatogram of the chimeric protein and the p28 purification using gel filtration and SDS-PAGE analysis after purification

(A) Chromatogram of the chimeric protein purification using gel filtration. The latter peak corresponds to the chimeric protein. Absorbance was monitored at 280 nm, at 25 °C, using an FPLC. (B) SDS-PAGE analysis of the chimeric protein purification using gel filtration. Lane 1; Undigested GST-chimeric protein before purification Lane 2; Digested GST-chimeric protein using enterokinase before purification Lane 3; the chimeric protein after purification Lane 4; Molecular weight marker Lane 5; GST after purification. Arrow indicated the chimeric protein. (C) Chromatogram of the p28 peptide purification using gel filtration. The latter peak corresponds to the p28 peptide. Absorbance was monitored at 280 nm, at 25 °C, using an FPLC. (D) SDS-PAGE analysis of the p28 peptide purification using gel filtration Lane 1; Molecular weight marker Lane 2; p28 peptide after purification Lane 3; GST after purification. Arrow indicated the p28 peptide.

**Figure 4:**
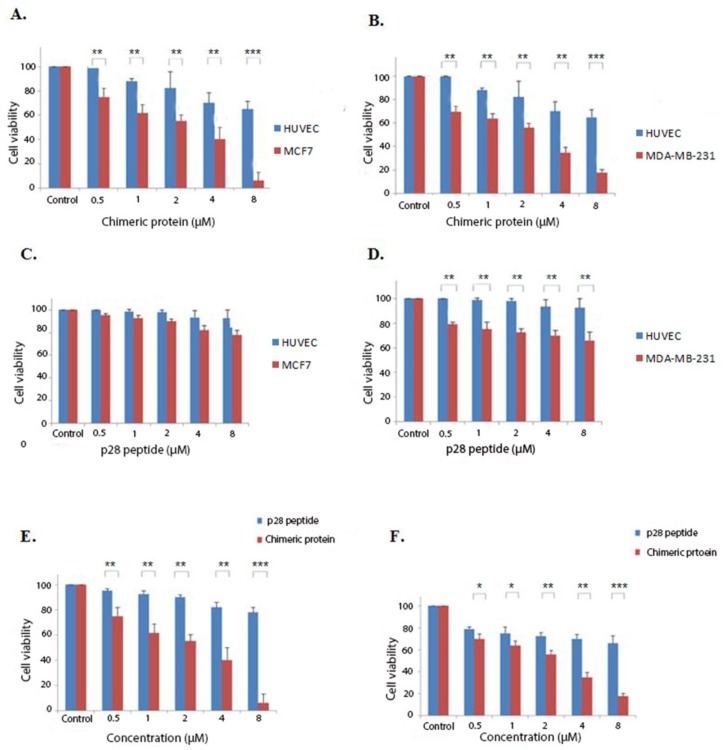
Cytotoxic effects of the chimeric protein and p28 peptide. (A) cytotoxic effects of the chimeric protein against MCF7 and HUVEC cells. (B) Cytotoxic effects of the chimeric protein against MDA-MB-231 and HUVEC cells. (C) Cytotoxic effects of the p28 against MCF7 and HUVEC cells. (D) Cytotoxic effects of the p28 against MDA-MB-231 and HUVEC cells. Cell viability was assessed using MTT assay after 48 h. (E) Incubation of the MCF7 cells with various concentrations of the p28 peptide and chimeric protein for 48 h. (F) Incubation of the MDA-MB-231 cells with various concentrations of the p28 peptide and the chimeric protein for 48 h. Cell viability was assessed using MTT assay after 48 h. Data were shown as Mean ± SD, n = 3. Percentage cell viability and comparison between data sets performed using ANOVA. *p < 0.05, **p < 0.01 and ***p < 0.001 indicates statistically significant differences between the means of values obtained with MTT assay

**Figure 5:**
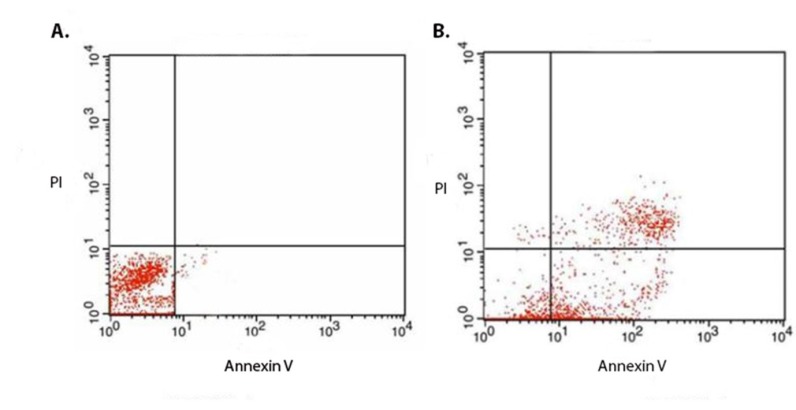
(A) Flow cytometric analysis of the non-treated and (B) treated cells for evaluation of the mechanism of the chimeric protein induced cell death. The MCF7 cells were treated with IC50 (1.88 µM) of the chimeric protein for 4 h. Annexin V and PI staining analysis of the MCF7 cells showed the induction of apoptosis in the treated cells. In case of untreated control cells, result showed 98.0% non- stained cells. However, for the treated MCF7 cells, the amount of the non-stained cells was 15.26% while the annexin V stained cells (early apoptotic cell) accounted for about 43% ± 6.36% (n = 3)

**Table 1:** Assessment of the pro- and anti- apoptotic factors transcription by Real Time RT-PCR. Results are represented as increased or decreased fold

**Pro- Apoptotic**	**Fold increase/decrease**	**Anti-Apoptotic**	**Fold increase/decrease**
AIF	4.26	Bcl2	-2.18
Bax	2.4		
FAS	0.9		
DR4	1.1		
Casp3	2.3		

*Evaluation of cell death mechanism by flow cytometry*

To assess the cell death mechanism induced by the chimeric protein using Flow cytometry, MCF7 cells were seeded in wells of six-well plates and treated with the IC_50_ concentration of the chimeric protein and subjected to staining with the Annexin V/PI according to instructions of the Annexin-V-FLUOS Staining kit (Roche, Germany). 

*Evaluation of pro- and anti-apoptotic gene transcriptions*

In order to further evaluate the mechanism of apoptosis induced by the chimeric protein, Real Time PCR analysis of mRNA expression of some pro- and anti-apoptotic genes was performed using Apoptosis RT Array™ kit (NanoCinna, Iran) according to the manufacturer’s instructions. 

The mRNA expression of GAPDH was considered as a housekeeping gene. Briefly, following treatment of MCF7 cells with the IC_50_ concentration of the chimeric protein, total cellular RNA was isolated using RNeasy™ Mini Kit (Qiagen, Germany) followed by DNAse treatment and subjected to cDNA synthesis using RevertAid™ First Strand cDNA Synthesis Kit (Thermo Scientific, USA). Next, the prepared cDNA was used for Real-time RT-PCR analysis by AB Applied BioSystems (Thermo Fisher Scientific, USA). 

*Statistical analyses*

Data were expressed as a mean ± standard error. The SPSS 20 software was used for statistical analysis. Statistical significance was determined by one-way ANOVA with Tukey′s post-hoc test. Differences were considered statistically significant when *p *< 0.05.

## Results

*Cloning, protein production, and western blot analysis*

Double digestion of the recombinant plasmids obtained following ligation and transformation of the pGEX-p28-NRC and pGEX-p28 revealed corresponding bands of about 230 bp and 110 bp, relatively, confirming the fidelity of cloning of the fragments. In addition, DNA sequencing also authenticated the cloning procedure. Next, an expression condition of temperature 37 °C, IPTG concentration of 1 mM, incubation time 6 h, and temperature = 37 °C, IPTG concentration of 1 mM, and incubation time 45 min were selected as best conditions for expression GST-p28 and GST-p28-NRC, respectively. Then, affinity chromatography using glutathione resin was used for purification of the GST-tagged chimeric protein. The purified fractions were analyzed by SDS-PAGE (Figures 1A and 1B) and confirmed by Western blotting (Figure 1C). The best condition for enterokinase cleavage was determined to be 16 h of incubation at 4 °C, in a buffer containing 20 mM Tris-HCl, pH 7.4, 200 mM NaCl, and 2 mM CaCl_2_ (Figures 2A and 2B). Gel filtration chromatography successfully separated the GST tag from chimeric protein and p28 peptide after the enterokinase cleavage steps, as shown by the diverse peaks in the chromatogram (Figures 3A and 3C). The related Coomassie stained SDS-PAGE confirmed high recovery and purity of the chimeric protein (Figure 3B) and p28 peptide (Figure 3D). Finally, the chimeric protein or the p28 peptide containing fractions were pooled, concentrated, and subjected to specific and non-specific cytotoxic studies.

*Cytotoxicity studies*

According to the initial preliminary studies, tests were performed with concentrations of 0.5-8 µM of the chimeric protein for 48 h. MTT assays were conducted on HUVEC, MCF7, and MDA-MB-231 cells treated with the chimeric protein and p28 peptide to determine their cytotoxicity against cancer cell lines. As it is shown by Figures 4A-4F, the chimeric protein killed MCF7 and MDA MB-231 human breast cancer cells in a dose-dependent manner with the IC_50_ values of 1.88 and 1.89 µM on the MCF7 and MDA MB-231 cells, respectively.

*Determination of cell death mechanism by Flow cytometry*

In order to identify cell death mechanism, MCF7 cells were treated with 3 µM of the chimeric protein, followed by staining with FITC coupled annexin V and propodium iodide (PI) and analyzed by Flow cytometry. In the case of untreated control cells, the result showed 98.0% non-stained cells. However, for the treated MCF7 cells, the amount of the non-stained cells was 15.26% while the annexin V stained cells (early apoptotic cell) accounted for about 43% ± 6.36% (n = 3) (Figure 5). This confirmed that cell death mechanism induced the MCF7 cells by the chimeric protein is via apoptosis.

*Evaluation of pro- and anti-apoptotic genes*

Following confirmation of the induced cell death mechanism to be apoptosis, variation in transcription of different pro- and anti-apoptotic proteins were evaluated by real-time RT-PCR. As it is shown in Table 1, the expression of the AIF, Bax, and caspase 7 pro-apoptotic genes was significantly increased, while no significant alteration was detected in the expression rates of Fas and DR4 pro-apoptotic proteins. On the other hand, the expression rate of the Bcl2 anti-apoptotic gene was significantly reduced to about more than 2 fold less expression when compared to the normal non-treated cells.

## Discussion

Due to the many drawbacks of non-targeted chemotherapeutics used for cancer treatment, it is a reinforcing need to develop targeted medications, which are able to destroy cancer cells selectively. Beside, anticancer therapy aims to find an approach to restrict side effects to the minimum, unaffected by common mechanisms of chemoresistance, and kill dormant and slow-growing cells. Chimeric peptide and protein have recently emerged as a potentially interesting anticancer agent because of their ability to kill cancer cell selectively and efficiently (13). In this study, we design a new chimeric protein and showed this protein is cytotoxic for breast cancer cell lines. 

In the previous study, because of the toxicity of NRC peptide against its expression in *Escherichia coli (E. coli) *strain, His-tag and Thioredoxin-tag were evaluated for the expression of the chimeric protein (14). Due to low yield after tags cleavage using enterokinase enzyme, in this study, we evaluated GST tag for expression of the chimeric protein. Among them, the GST tag showed to be more effective for obtaining a higher yield of chimeric protein after tag cleavage. The capability of GST as a fusion tag for antibacterial peptide expression was also demonstrated by another study (15).

Potential improved cytotoxic effects of the chimeric protein on killing breast cancer cell lines were evaluated by comparing the cytotoxicity of the chimeric protein and the p28 peptide. As shown by the findings of the MTT assay, the chimeric protein had greater cell toxicity than the p28 peptide, suggesting its enhanced effects on breast cancer cell lines. In our study, the obtained IC_50_ for the chimeric protein against MCF7 cells was about 1.88 µM whereas the IC_50_ of the p28 peptide was 23 µM. Song-Hee HAM *et al*. expressed a chimeric protein comprising of gelonin and F peptide. Their finding indicated that this chimeric protein had significantly higher cytotoxicity against cancer cells compared with gelonin alone (16). Another example of cytotoxic chimeric protein is DT386-BR2 with promising cytotoxic effects suggesting that it can be a candidate for targeted cancer therapy (17).

There was a significant difference between the cytotoxicity of the chimeric protein against normal and cancer lines. This difference suggested that chimeric protein can selectively enter breast cancer cell lines. Selective entrance indicates that p28 can be used as a targeting peptide in a chimeric peptide and protein design. This strategy can be used for another nonspecific killing moiety to reduce their nonspecific toxicity. For example, Shafiee *et al*. used BR2 peptide for targeted delivery of diphtheria toxin to cancer cells (17). 

The development of multidrug resistance cancer cells after chemotherapy is a big issue in the cancer therapy (18). The sensitivity of two cancer lines was the almost same because the NRC peptide cell death mechanism is non-specifically as mitochondrial membrane damage and ROS production (12). This finding and significant difference between cancer and normal cell lines indicate the possibility that chimeric protein kills multidrug-resistant cancer cells selectively. On the other hand, the mechanism of cell death of this chimeric protein is not related to cell growth. So, this protein is likely will effective against slow-growing cancer cells. 

The results of flow cytometric analysis clearly establish the efficient induction of apoptotic cell death in MCF7 cells by the chimeric protein. The Real-Time PCR assay showed downregulation of Bcl-2 expression and the upregulation of Bax and casapase-7 expression, which induces outer mitochondrial membrane permeabilization and the loss of mitochondrial potential in the apoptotic death. AIF is a factor of caspase-independent peripheral chromatin condensation and large-scale DNA fragmentation (19). Upregulation of this protein has been reported by other studies (20). Our results revealed 4.26 fold upregulation in the expression level of AIF mRNA following treatment of the MCF7 cells with the chimeric protein. Therefore, these results indicated that the chimeric protein exerts its cytotoxic effects on MCF7 breast cancer cells through mitochondrial caspase-dependent and –independent apoptotic pathways. The multifaceted nature of malignant neoplasms suggests that drugs using different pathways to induce cell death may be more effective than single-mechanism therapies (21). This two-pronged action of the chimeric protein gives it an additional advantage over traditional cytotoxic chemotherapeutic agents and overcoming to the tumor resistance. 

The main challenge of using chimeric protein is to design them in a way that different moiety does not intervene each other’s function. Bioinformatics tools as a fast growing field can be used for chimeric protein design. We previously design this chimeric protein using homology modeling and molecular dynamic simulation. According to the results of bioinformatics studies, they were fused to each other via a rigid linker consisting of (PA)_5_P linker (22). The enhanced cytotoxicity results of the chimeric protein confirm that bioinformatics tools can be used for chimeric protein design.

## Conclusion

In conclusion, in the present study, we designed and evaluated the applicability of a novel chimeric protein for anti-breast cancer efficacy *in-vitro*. The chimeric protein and the p28 peptide were successfully produced in *E. coli* Consistent with the findings from the MTT assays, chimeric protein had synergistic anti-breast cancer cells effects when compared with the administration of p28 alone. Overall, these data suggest that p28 conjugation with the NRC peptide improves its anti-cancer activity and this combinatorial therapy might be an alternative for targeted therapy of cancer. 
